# Prognostic interaction between *ASXL1* and *TET2* mutations in chronic myelomonocytic leukemia

**DOI:** 10.1038/bcj.2015.113

**Published:** 2016-01-15

**Authors:** M M Patnaik, T L Lasho, P Vijayvargiya, C M Finke, C A Hanson, R P Ketterling, N Gangat, A Tefferi

**Affiliations:** 1 Division of Hematology, Department of Medicine, Mayo Clinic, Rochester, MN, USA; 2 Department of Laboratory Medicine, Mayo Clinic, Rochester, MN, USA

## Abstract

Mutations involving epigenetic regulators (*TET2*~60% and *ASXL1*~40%) and splicing components (*SRSF2*~50%) are frequent in chronic myelomonocytic leukemia (CMML). On a 27-gene targeted capture panel performed on 175 CMML patients (66% males, median age 70 years), common mutations included: *TET2* 46%, *ASXL1* 47%, *SRSF2* 45% and *SETBP1* 19%. A total of 172 (98%) patients had at least one mutation, 21 (12%) had 2, 24 (14%) had 3 and 30 (17%) had >3 mutations. In a univariate analysis, the presence of *ASXL1* mutations (*P*=0.02) and the absence of *TET2* mutations (*P*=0.03), adversely impacted survival; while the number of concurrent mutations had no impact (*P*=0.3). In a multivariable analysis that included hemoglobin, platelet count, absolute monocyte count and circulating immature myeloid cells (Mayo model), the presence of *ASXL1* mutations (*P*=0.01) and absence of *TET2* mutations (*P*=0.003) retained prognostic significance. Patients were stratified into four categories: *ASXL1wt/TET2wt* (*n*=56), *ASXL1mut/TET2wt* (*n*=31), *ASXL1mut/TET2mut* (*n*=50) and *ASXL1wt/TET2mut* (*n*=38). Survival data demonstrated a significant difference in favor of *ASXL1wt/TET2mut* (38 months; *P*=0.016), compared with those with *ASXL1wt/TET2wt* (19 months), *ASXL1mut/TET2wt* (21 months) and *ASXL1mut/TET2mut* (16 months) (*P*=0.3). We confirm the negative prognostic impact imparted by *ASXL1* mutations and suggest a favorable impact from *TET2* mutations in the absence of *ASXL1* mutations.

## Introduction

Gene mutations are common (>90%) in chronic myelomonocytic leukemia (CMML) and involve epigenetic regulators (*TET2*~60% and *ASXL1*~40%), spliceosome components (*SRSF2*~50%) and cell signaling (*RAS*~30% and *CBL*~15%).^1, 2, 3, 4^ Mutations involving *ASXL1, TET2, RUNX1, CBL, SRSF2, RAS* and *IDH2* have demonstrated prognostic relevance on univariate survival analyses.^1, 5, 6^ However, on multivariable analyses that have included additional CMML relevant factors, only *ASXL1* mutations (frameshift and nonsense) have been shown to be prognostically detrimental.^1, 2^ This has led to the incorporation of *ASXL1* mutations into molecular prognostic models such as the Molecular Mayo Model and the Groupe Francais des Myelodysplasies model.^1, 2^

*TET2* mutations (chromosome 4q24) are frequent and are thought to be the driver mutations in CMML.^7^ TET2 catalyzes the conversion of 5-methyl-cytosine to 5-hydroxymethyl-cytosine, regulating methylation and transcription.^8^ The prognostic relevance of *TET2* mutations remains unclear with some studies demonstrating favorable,^9^ unfavorable^10^ and no impact^1^ on overall survival (OS). *In* *vitro* studies have shown that *ASXL1* mutations enhance the de-ubiquitinase activity of the ASXL1–BAP1 (BRCA associated protein 1) complex, which then cooperates with loss of TET2 to skew towards myeloid development.^11^ However, the mechanisms behind this effect and the prognostic interplay between *TET2* and *ASXL1* mutations remain unknown.

In the current study, we used a 27-gene panel assay to: (i) identify additional prognostically-relevant mutations in CMML, (ii) to determine if the number of mutations carries prognostic relevance and (iii) to study the prognostic interplay between *TET2* and *ASXL1* mutations.

## Materials and methods

One-hundred and seventy five patients with CMML were included in the study. All patients had bone marrow biopsies and cytogenetic studies performed at diagnosis. The diagnosis of CMML, including subclassification into CMML-1 or CMML-2, and leukemic transformation were according to the 2008 World Health Organization criteria.^12^ Risk stratification was per the Mayo-French cytogenetic system,^13^ the Mayo model,^14^ the Groupe Francais des Myelodysplasies model^1^ and the Molecular Mayo model.^2^ Twenty-seven gene panel targeted capture assays were carried out on bone marrow DNA specimens obtained at diagnosis for the following genes: *TET2, DNMT3A, IDH1, IDH2*, *ASXL1, EZH2, SUZ12*, *SRSF2, SF3B1, ZRSR2, U2AF1*, *PTPN11, Tp53, SH2B3, RUNX1, CBL, NRAS, JAK2, CSF3R, FLT3, KIT, CALR, MPL*, *NPM1, CEBPA, IKZF* and *SETBP1*.

Paired-end indexed libraries were prepared from individual patient DNA in the Mayo Clinic Genomic Sequencing Core Laboratory using the NEBNext Ultra Library prep protocol on the Agilent Bravo liquid handler (NEB, Ipswich, MA, USA/Agilent Technologies Inc., Santa Clara, CA, USA). Capture libraries were assembled according to the Nimblegen standard library protocol (Roche Nimblegen, Inc., Basel, Switzerland). A panel including the regions of 27 heme-related genes was selected for custom target capture using the Agilent SureSelect Target Enrichment Kit (Agilent Technologies Inc, Santa Clara, CA, USA). Capture libraries were pooled at equimolar concentrations and loaded onto paired end flow cells at concentrations of 7–8 pM to generate cluster densities of 600 000–800 000/mm^2^ following Illumina's standard protocol using the Illumina cBot and HiSeq Paired end cluster kit version 3, in batches of 48 samples per lane (Illumina Incorporated, San Diego, CA, USA). The flow cells were sequenced as 101 × 2 paired end reads on an Illumina HiSeq 2000 using TruSeq SBS sequencing kit version 3 (Illumina Incorporated) and HiSeq data collection version 2.0.12.0 software (Illumina Incorporated). Base-calling was performed using Illumina's RTA version 1.17.21.3 (Illumina Incorporated).

Genesifter software was utilized (PerkinElmer, Danvers, MA, USA) to analyze targeted sequence data. Reads from the sequencing in fastq format were aligned using the Burrows-Wheeler Aligner against the genomic reference sequence for Homo sapiens (Build 37.2; NCBI http://www.ncbi.nlm.nih.gov/). An additional alignment, post-processing set of tools were then used to do local realignment, duplicate marking and score recalibration to generate a final genomic aligned set of reads. Nucleotide variants were called using the Genome Analysis Toolkit (GATK -Broad Institute, Cambridge, MA, USA) that identified single nucleotide and small insertion/deletion events using default settings. Specific variants were deemed as mutations if they were associated with a heme malignancy (as identified by COSMIC database), or if they have not been associated with a single nucleotide polymorphism database.

Based on prior observations, only frame shift and nonsense *ASXL1* mutations were considered pathogenic.^2, 14^ For *TET2*, frame shift, nonsense, missense, insertions and deletions were considered pathogenic. Previously annotated single nucleotide polymorphisms (http//www.hapmap.org) in all the aforementioned genes were considered nonpathogenic.

All statistical analyses considered parameters obtained at time of referral to the Mayo Clinic, which in most instances coincided with time of bone marrow biopsy. Differences in the distribution of continuous variables between categories were analyzed by either Mann–Whitney (for comparison of two groups) or Kruskal–Wallis (comparison of three or more groups) test. Patient groups with nominal variables were compared by the chi-square test. Overall survival was calculated from the date of first referral to date of death (uncensored) or last contact (censored). Leukemia-free survival (LFS) was calculated from the date of first referral to date of leukemic transformation (uncensored) or death/last contact (censored). Overall and LFS curves were prepared by the Kaplan–Meier method and compared by the log-rank test. Cox proportional hazard regression model was used for multivariable analysis. *P* <0.05 were considered significant. The Stat View (SAS Institute, Cary, NC, USA) statistical package was used for all calculations.

## Results

Among the 175 study patients, 115 (66%) were males with a median age of 70 years (range, 18–90). One hundred and forty-six (83%) patients were subclassified as CMML-1 and the remainder had CMML-2. At a median follow-up of 23 months, 146 (83%) deaths and 25 (14%) leukemic transformations were documented. Median survivals were 24 months for CMML-1 and 16 months for CMML-2 (*P*=0.38). Cytogenetic risk stratification was carried out using the Mayo-French cytogenetic model,^13^ with the following distribution: 118 (78%) low, 21 (10%) intermediate and 18 (12%) high risk. Overall risk stratification was based on Mayo prognostic model:^14^ 25% high, 32% intermediate and 43% low risk; Molecular Mayo Model:^2^ 30% high, 30% intermediate-2, 31% intermediate-1 and 9% low risk; and the Groupe Francais des Myelodysplasies model:^1^ 19% high, 37% intermediate and 44% low risk. Baseline laboratory values and risk stratification are detailed in Table 1.

**Figure 1:**
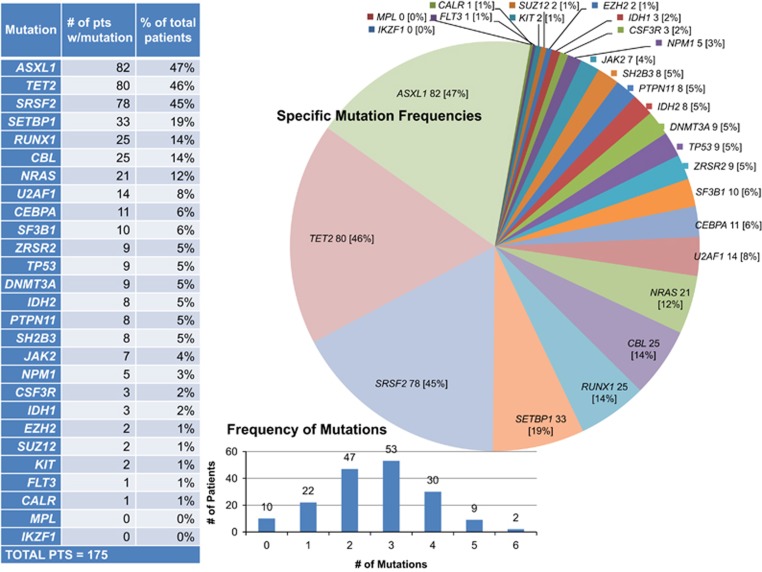
Spectrum and frequency of gene mutations in 175 Mayo clinic patients with WHO defined chronic myelomonocytic leukemia.

Mutational frequencies were as follows: *TET2* 46%, *ASXL1* 47%, *SRSF2* 45%, *SETBP1* 19%, *CBL* 14%, *RUNX1* 14%, *NRAS* 12%, *U2AF1* 8%, *SF3B1* 6%, ZRSR2 6%, *Tp53* 5%, *DNMT3A* 5%, *IDH2* 5%, *PTPN11* 5%, *SH2B3* 5%, *JAK2*V617F 4%, *NPM1* 3%, *CSF3R* 2%, *IDH1* 2%, *EZH2* 1%, *SUZ12* 1%, *KIT* 1%, *FLT3* 1% and *CALR* 1% (Figure 1 and Table 1). No mutations were detected in *MPL* or *IKZF*. One hundred and seventy two patients (98%) had at least one mutation, 21 (12%) had 2, 24 (14%) had 3, 20 (11%) had 4, 9 (5%) had 5; while one (1%) patient had 6 concurrent mutations (Figure 1).

**Figure 2:**
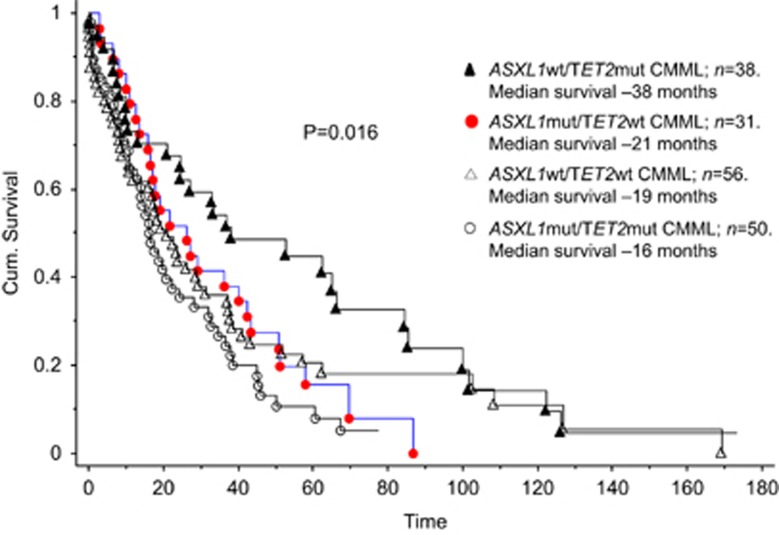
Survival data for 175 patients with chronic myelomonocytic leukemia stratified by *ASXL1* and *TET2* mutational status.

In a univariate survival analysis that included the aforementioned mutations, only the presence of *ASXL1* mutations (*P*=0.01), absence of *TET2* mutations (*P*=0.005) and presence of *DNMT3A* mutations (*P*=0.02) were associated with inferior survival. The number of concurrent mutations per patient did not affect outcome (*P*=0.3). In a multivariable analysis, the presence of *ASXL1* (*P*=0.01) and the absence of *TET2* (*P*=0.03) mutations retained their negative prognostic impact. In order to determine the prognostic interaction between these two mutations, patients were stratified into four mutational categories: *ASXL1wt/TET2wt* (*n*=56), *ASXL1mut/TET2wt* (*n*=31), *ASXL1mut/TET2mut* (*n*=50) and *ASXL1wt/TET2mut* (*n*=38). Survival data in these four groups showed significant difference in favor of *ASXL1wt/TET2mut* (median survival 38 months; *P*=0.016), compared with those with *ASXL1wt/TET2wt* (19 months), *ASXL1mut/TET2wt* (21 months) and *ASXL1mut/TET2mut* (16 months); there was no significant difference in survival among the latter three groups (*P*=0.3) (Figure 2).

In multivariable analysis, presence of *ASXL1* (*P*=0.01) and absence of *TET2* mutations (*P*=0.003) remained significant when risk factors used in the Mayo prognostic model (hemoglobin <10 gm/dl, absolute monocyte count >10x10(9)/L, platelet count <100x10(9)/L, presence of circulating immature myeloid cells) were added to the model;^14^ the same was true for *ASXL1wt/TET2mut* (*P*=0.036). In a separate multivariable analysis that included the Mayo prognostic model as a single variable along with presence of *ASXL1* and absence of *TET2* mutations or absence of *ASXL1wt/TET2mut* mutational status, the respective hazard ratios were 1.4 (95% CI 1.07–2.1; *P*=0.012), 1.5 (95% CI 1.07–2.1; *P*=0.03) and 1.8 (95% CI 1.2–2.7; *P*=0.001). On a univariate analysis, LFS was worse in *ZRSR2*-mutated cases (*P*=0.03). This relevance, however, was lost on a multivariable analysis that included circulating blasts (*P*=0.01) and high risk karyotype (*P*=0.03).

## Discussion

Clonal cytogenetic abnormalities are seen in ~30%,^13, 15^ while gene mutations are seen in >90% of patients with CMML.^1, 2, 16^ These mutations can broadly be classified into the following categories: (i) mutations involving epigenetic regulator genes: *TET2 (~60%), DNMT3A, IDH1,* and *IDH2* (*IDH* mutations <10%); (ii) mutations involving histone modification and chromatin regulation: *ASXL1 (~40%)* and *EZH2* (<5%); (iii) mutations involving the splicing machinery: *SF3B1, SRSF2 (~50%), U2AF1 and ZRSR2;* (iv) mutations involving DNA damage response genes: *Tp53 (~1%) and PHF6;* (v) mutations in transcription factors and signal transduction pathways: *JAK2, KRAS, NRAS (RAS~30%), CBL (~10–15%), FLT3, RUNX1(~15%)* and mutations such as *SETBP1* (~15%).^1, 2, 16, 17, 18, 19^ Of these, mutations involving *TET2* (~60%), *SRSF2* (~50%), *ASXL1* (~40%) and the *RAS* pathway (~30%) are most frequent, with only frameshift and nonsense *ASXL1* mutations independently impacting OS.^1, 2^

The *ASXL1* (additional sex combs like 1) gene (chromosome 20q11) regulates chromatin by interacting with the polycomb-group repressive complex proteins (PRC1 and PRC2).^20^ Histone 2A lysine 119 (H2AK119Ub) and H3K27me3 play synergistic roles in PRC-mediated gene repression.^11, 21^ Abdel-Wahab *et al.*^21^ demonstrated that *ASXL1* mutations resulted in loss of PRC2-mediated H3K27 tri-methylation, while Balasubramani *et al.*^11^ demonstrated that *ASXL1* truncations conferred enhanced activity on the ASXL1–BAP1 complex. This complex results in global erasure of H2AK119Ub and depletes H327Kme3, promoting dysregulated transcription. The current study once again demonstrates the frequent occurrence of *ASXL1* mutations (45%) in CMML and confirms the adverse prognostic impact imparted by frameshift and nonsense mutations on OS.

*TET2* (t*en-eleven* trans*location* (TET) oncogene family member 2) is a member of the TET family of proteins.^22^ Although *TET2* mutations are widely prevalent in CMML, thus far, they have not been shown to independently impact either OS or LFS.^1^ In the current study, *TET2* mutations were seen in 46% of CMML patients and the absence of *TET2* mutations negatively impacted OS. Additionally, the presence of clonal *TET2* mutations, in the absence of clonal *ASXL1* mutations (*ASXL1wt/TET2mut*), had a favorable impact on OS. The mechanism behind this association is unclear. In MDS and younger patients with CMML (age <65 years), the presence of clonal *TET2* mutations, in the absence of clonal *ASXL1* mutations, have been associated with response to hypomethylating agents (5-azacitidine and decitabine).^5, 23^ Treatment data on our cohort of patients were incomplete and it is currently unknown as to whether this favorable impact was an effect of better responses to hypomethylating agents or not.

Approximately, 80% of patients with MDS have one or more oncogenic driver mutations (*SF3B1*~24%, *TET2*~22%, *SRSF2*~15% and *ASXL1*~15%).^4^ In a large study (*n*=738), Papaemmanuil *et al.*^4^ demonstrated that driver mutations had an equivalent prognostic significance and LFS steadily declined as the number of driver mutations increased. 78% had at least one oncogenic mutation, while 43% had 2 or 3 and 10% had 4–8 mutations. Variants of unclear significance in oncogenic genes such as *ASXL1* also adversely impacted outcomes. In the current study, 98% of the CMML patients had at least one mutation, 12% had 2, 14% had 3 and 17% had >3 mutations. The number of oncogenic mutations in CMML did not impact either the LFS or OS.

In summary, nearly all patients with CMML express one or more myeloid neoplasm-relevant mutations. Similar to prior studies, the three most frequent mutations include *TET2*, *ASXL1* and *SRSF2*.^1, 2^ Unlike in MDS, survival outcomes in CMML were not affected by the number of concurrent driver mutations. We confirm the negative prognostic impact on OS imparted by *ASXL1* mutations^1, 2^ and also suggest a favorable prognostic impact from *TET2* mutations, unless accompanied by *ASXL1* mutations. These findings need validation in a larger data set.

## Figures and Tables

**Table 1:** Clinical and laboratory features and subsequent events in 175 patients with World Health Organization defined chronic myelomonocytic leukemia, stratified by *ASXL1* and *TET2* mutational status

*Variable*	*All patients with CMML*	*CMML patients with ASXL1 mutations*	*CMML patients with TET2 mutations*
	*(*n=*175)*	*(*n=*82)*	*(*n=*80)*
Age in years; median (range)	70 (18–90)	69 (27–86)	70 (40–90)
Males; *n* (%)	116 (66)	59 (72.0)	56 (70)
Hemoglobin g/dL; median (range)	10.5 (6.4–16.9)	10.5 (6.4–15.1)	11.5 (6.8–15.3)
WBCx10^9^/L; median (range)	11.1 (1.5 –264.8)	13.1 (1.8–264)	9.3 (1.8–264)
ANCx10^9^/L; median (range)	5.2 (0–151)	5.7 (0–151)	5.2 (0.2–142.9)
AMCx10^9^/L; median (range)	2.3 (0.3–40)	2.6 (0.6–40)	2 (0.34–40)
ALCx10^9^/L; median (range)	1.5 (0–22)	1.6 (0.4–22)	1.4 (0–22)
Plateletsx10^9^/L; median (range)	87 (10–585)	82 (10–339)	77 (10–585)
Presence of circulating immature myeloid cells; *n* (%)	84 (48)	47 (57.3)	29 (36.3)
PB blast % median (range)	0 (0–19)	0 (0–19)	0 (0–12)
BM blast % median (range)	3 (0–19)	4 (0–19)	2 (0–16)
			
*WHO morphological subtype;* n *(%)*			
* *CMML-1	146 (83)	67 (81.7)	75 (93.8)
* *CMML-2	29 (17)	15 (18.3)	5 (6.1)
			
*Mutational analysis*			
* IKZF*	0 (0)	0 (0)	0 (0)
* PTPN11*	8 (4.5)	5 (6)	0 (0)
* SH2B3*	8 (4.5)	5 (6)	6 (7.5)
* SUZI12*	2 (1.1)	1 (1.2)	1 (1.25)
* ZRSR2*	9 (5.1)	6 (7.3)	7 (8.75)
* CALR*	1 (0.57)	0 (0)	0 (0)
* CBL*	25 (14.3)	14 (17)	12 (15)
* CEBPA*	11 (6.3)	6 (7.3)	4 (5)
* CSF3R*	3 (1.7)	2 (2.4)	1 (1.25)
* DNMT3A*	9 (5.1)	3 (3.7)	2 (2.5)
* EZH2*	2 (1.1)	1 (1.2)	1 (1.25)
* FLT3*	1 (0.57)	1 (1.2)	0 (0)
* IDH1*	3 (1.7)	2 (2.4)	0 (0)
* IDH2*	8 (4.5)	5 (6)	1 (1.25)
* JAK2*	7 (4)	4 (4.9)	1 (1.25)
* KIT*	2 (1.1)	1 (1.2)	1 (1.25)
* MPL*	0 (0)	0 (0)	0 (0)
* NPM1*	5 (2.9)	0 (0)	1 (1.25)
* NRAS*	21 (12)	12 (14.6)	9 (11.25)
* RUNX1*	25 (14.3)	13 (15.9)	10 (12.5)
* SETBP1*	33 (18.9)	23 (28)	11 (13.75)
* SF3B1*	10 (5.7)	1 (1.2)	5 (6.25)
* SRSF2*	93 (53.1)	39 (47.6)	41 (51.25)
* Tp53*	9 (5.1)	1 (1.2)	1 (1.25)
* U2AF1*	14 (8)	11 (13.4)	2 (2.5)
* ASXL1*	82 (46.9)	N/A	31 (38.75)
* TET2*	80 (45.7)	31 (37.8)	N/A
			
*Mayo-French cytogenetic risk stratification;* n *(%)*			
Low	118 (78)	51 (70)	66 (83)
Intermediate	21 (10)	11 (14)	6 (8)
High	18 (12)	9 (16)	1 (9)
			
*MD Anderson prognostic risk categories;* n *(%)*			
Low	90 (51.4)	35 (42.7)	53 (66.25)
Intermediate-1	41 (23.4)	22 (26.8)	13 (16.25)
Intermediate-2	35 (20)	21 (25.6)	14 (17.5)
High	9 (5.1)	4 (4.9)	0 (0)
			
*Mayo model prognostic risk categories;* n *(%)*			
Low	76 (43.4)	28 (34.1)	40 (50)
Intermediate	56 (32)	32 (39)	28 (35)
High	43 (24.6)	22 (26.8)	12 (15)
			
*Molecular Mayo Model risk categories;* n *(%)*			
Low	16 (9.1)	3 (3.66)	11 (13.75)
Intermediate-1	55 (31.4)	12 (14.6)	29 (36.25)
Intermediate-2	52 (29.7)	30 (36.6)	29 (36.25)
High	52 (29.7)	37 (45.1)	11 (13.75)
			
*GFM prognostic risk categories;* n *(%)*			
Low	77 (44)	17 (20.7)	46 (57.5)
Intermediate	65 (37.1)	40 (48.8)	20 (25)
High	33 (18.9)	25 (30.5)	14 (17.5)
Leukemic transformations; *n* (%)	25 (14.3)	13 (15.9)	11 (13.75)
Deaths; *n* (%)	146 (83.4)	71 (86.6)	62 (77.5)

Abbreviations: ALC, absolute lymphocyte count; AMC, absolute monocyte count; ANC, absolute neutrophil count; *ASXL1,* additional sex combs 1 gene; BM, bone marrow; CMML, chronic myelomonocytic leukemia; GFM, Groupe Francais des Myelodsyplasies; NA, not applicable; PB, peripheral blood; *SF3B1,* splicing factor 3B subunit 1; *SRSF2,* serine/arginine-rich splicing factor 2; *U2AF1,* U2 small nuclear RNA auxiliary factor 1; WBC, white blood cell count; WHO, World Health Organization.
